# Genomic editing in *Burkholderia multivorans* by CRISPR/Cas9

**DOI:** 10.1128/aem.02250-23

**Published:** 2024-02-02

**Authors:** Mirela R. Ferreira, Vasco Queiroga, Leonilde M. Moreira

**Affiliations:** 1 iBB-Institute for Bioengineering and Biosciences, Instituto Superior Técnico, Universidade de Lisboa, Lisbon, Portugal; 2 Associate Laboratory i4HB-Institute for Health and Bioeconomy, Instituto Superior Técnico, Universidade de Lisboa, Lisbon, Portugal; 3 Department of Bioengineering, Instituto Superior Técnico, Universidade de Lisboa, Lisbon, Portugal; University of Nebraska-Lincoln, USA

**Keywords:** *B. multivorans*, genome editing, CRISPR/Cas9, cystic fibrosis

## Abstract

*Burkholderia cepacia* complex bacteria have emerged as opportunistic pathogens in patients with cystic fibrosis and immunocompromised individuals, causing life-threatening infections. Because of the relevance of these microorganisms, genetic manipulation is crucial for explaining the genetic mechanisms leading to pathogenesis. Despite the availability of allelic exchange tools to obtain unmarked gene deletions in *Burkholderia*, these require a step of merodiploid formation and another of merodiploid resolution through two independent homologous recombination events, making the procedure long-lasting. The CRISPR/Cas9-based system could ease this constraint, as only one step is needed for allelic exchange. Here, we report the modification of a two-plasmid system (pCasPA and pACRISPR) for genome editing in *Burkholderia multivorans*. Several modifications were implemented, including selection marker replacement, the optimization of *araB* promoter induction for the expression of Cas9 and λ-Red system encoding genes, and the establishment of plasmid curing procedures based on the *sacB* gene or growth at a sub-optimal temperature of 18°C–20°C with serial passages. We have shown the efficiency of this CRISPR/Cas9 method in the precise and unmarked deletion of different genes (*rpfR*, *bceF*, *cepR*, and *bcsB*) from two strains of *B. multivorans*, as well as its usefulness in the targeted insertion of the *gfp* gene encoding the green fluorescence protein into a precise genome location. As pCasPA was successfully introduced in other *Burkholderia cepacia* complex species, this study opens up the possibility of using CRISPR/Cas9-based systems as efficient tools for genome editing in these species, allowing faster and more cost-effective genetic manipulation.

**IMPORTANCE:**

*Burkholderia* encompasses different species of bacteria, some of them pathogenic to animals and plants, but others are beneficial by promoting plant growth through symbiosis or as biocontrol agents. Among these species, *Burkholderia multivorans*, a member of the *Burkholderia cepacia* complex, is one of the predominant species infecting the lungs of cystic fibrosis patients, often causing respiratory chronic infections that are very difficult to eradicate. Since the *B. multivorans* species is understudied, we have developed a genetic tool based on the CRISPR/Cas9 system to delete genes efficiently from the genomes of these strains. We could also insert foreign genes that can be precisely placed in a chosen genomic region. This method, faster than other conventional strategies based on allelic exchange, will have a major contribution to understanding the virulence mechanisms in *B. multivorans*, but it can likely be extended to other *Burkholderia* species.

## INTRODUCTION

Members of the genus *Burkholderia* are common soil inhabitants, often associated with plants, either living freely in the rhizosphere or exhibiting a symbiotic lifestyle. They have vast biotechnological potential, producing important hydrolytic enzymes and other bioactive compounds and degrading recalcitrant pollutants (1). Yet, their use is limited because of the pathogenic potential of some strains for humans and other animals. Among these are the species of the *Burkholderia cepacia* complex (*Bc*c), a group of opportunistic pathogens capable of causing severe respiratory infections in patients with the genetic disease cystic fibrosis (CF) and in other immunocompromised patients (2). These bacteria show resistance to multiple antimicrobial agents and can be spread from patient to patient, leading to rapidly progressing and difficult-to-treat lung infections. *Burkholderia multivorans* and *Burkholderia cenocepacia* are two of the several genetically distinct species identified within the *Burkholderia cepacia* complex and the two most prevalent CF pathogens in this complex (3). Clinical outcomes among patients infected with *Bc*c bacteria are highly variable and unpredictable. This heterogeneity in prognosis remains inadequately explained, partly because of a lack of knowledge regarding some virulence factors that lead to pathogenesis (2, 4).

A valuable way to study the genetic mechanisms underlying virulence is through the comparison of the phenotype of a wild-type organism with one in which a specific gene has been knocked out. Therefore, experimental approaches for the generation of unmarked deletions based on allelic exchange are available for *Burkholderia* (5, 6). The more recent one, developed by Fazli and co-authors (6), uses Gateway cloning to introduce the flanking regions of a target gene into a suicide vector. Integrating this plasmid into the *Burkholderia* genome creates merodiploids, which are resolved by a second homologous recombination stimulated by the yeast homing endonuclease I-SceI expressed from a second plasmid. Finally, curing of this second plasmid is done in the presence of *pheS* gene whose product kills cells when grown in the presence of *p*-chlorophenyl alanine (6). Despite the success of this strategy in generating deletion mutants in *Burkholderia* species, the need to create merodiploids in one step and their resolution in a second step is time-consuming. This can be shortened by the use of CRISPR/Cas technology, as in this method no merodiploids are generated. Thus, CRISPR/Cas9 systems have been used for the genetic manipulation of several bacteria, including the *Burkholderia* genus. For example, a CRISPR-associated transposase was used in *Burkholderia thailandensis* to disrupt genes by the targeted insertion of a transposon (7). In another study, a two plasmid-based CRISPR/Cas9 system (8) was used to create small deletions in *Burkholderia contaminans*-specific genes exploring the non-homologous end-joining mechanism to repair mutations (9). However, none of these methods generated full gene deletion. Thus, the development of alternative CRISPR/Cas9-based systems able to create gene knock-out and knock-in, allowing for more efficient and less laborious genome editing techniques, is needed.

The CRISPR/Cas9 system comprises a single guide RNA (sgRNA) and the Cas9 endonuclease. The sgRNA contains the spacer, a 20-bp sequence based on the desired target sequence, which forms a complex with the Cas9 nuclease, guiding it to the specific genomic locus through a base pairing of the spacer with the genomic DNA. To successfully cleave the target sequence, Cas9 requires a short DNA sequence named protospacer-adjacent motif (e.g., 5′-NGG-3′ for *Streptococcus pyogenes* Cas9) that must be present immediately downstream of the target site. After binding, the Cas9 nuclease cuts both strands of the DNA at the target site, generating a double-stranded break (DSB). If not repaired, DSBs can be lethal, and thus cells need to repair these breaks through one of two mechanisms: the non-homologous end joining repair pathway, which typically leads to random DNA insertions or deletions or the homology-directed repair pathway (HDR), in which a donor DNA template with flanking homologous regions is used to replace the damaged DNA sequence through homologous recombination (8). CRISPR/Cas9 systems associated with HDR have already been exploited to generate sequence-specific gene editing in several bacterial species, such as *Escherichia coli*, *Streptococcus pneumoniae*, *Pseudomonas aeruginosa*, to mention a few (8–10).

Since bacteria rely primarily on the HDR pathway to repair their DSBs, only cells that undergo homologous recombination can survive. However, some bacteria have poor intrinsic homologous recombination capacity and require the use of methods to increase HDR efficiency. Phage recombination systems, such as the λ-Red system, have shown significant results in promoting homologous recombination in different bacteria (8, 10–13). In a previous study, Chen and co-authors (10) combined the λ-Red system with CRISPR/Cas9 to develop a tool for efficient genome editing in *Pseudomonas aeruginosa*. In this tool shown in Fig. S1, the pCasPA plasmid expresses both the Cas9 endonuclease and the λ-Red system proteins Exo, Gam, and Bet, and their expression is induced by L-arabinose. The pACRISPR plasmid expresses the sgRNA and carries the repair template necessary for HDR. Both plasmids contain the counter-selectable marker *sacB* gene, which confers sensitivity to sucrose, facilitating plasmid curing after genome editing.

This work describes the modifications to the previously described two-plasmid system (pCasPA/pACRISPR) for CRISPR/Cas9-mediated genome editing toward its successful implementation in *Burkholderia multivorans* strains’ genetic manipulation. Several gene knockouts and the locus-specific chromosomal integration of the *gfp* gene were achieved, simplifying the genetic manipulation tools in these microorganisms.

## RESULTS

### Mobilization of plasmid pCasPA into several strains of *Burkholderia*

**Fig 1:**
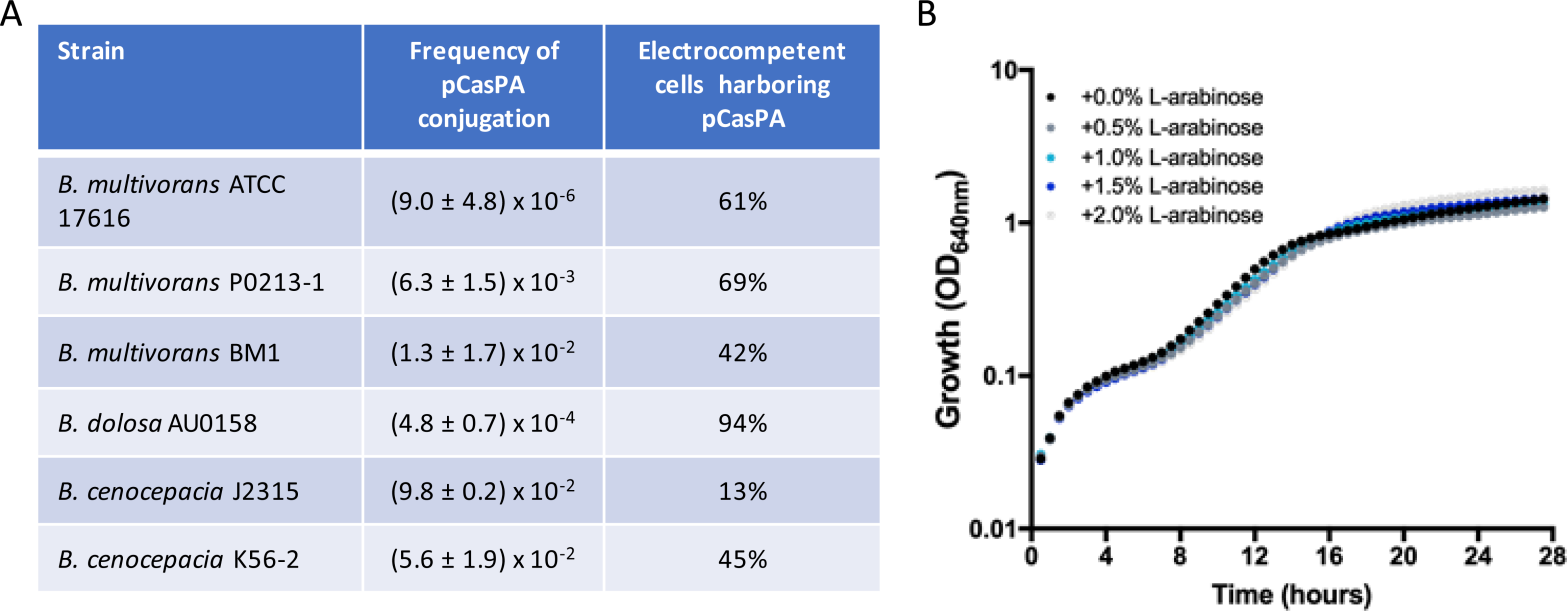
Transformation and maintenance of pCasPA into *Burkholderia* strains. (**A**) Plasmid pCasPA was mobilized into *Burkholderia* by triparental mating and the frequencies of conjugation are presented. After preparing L-arabinose-induced electrocompetent cells from single colonies harboring pCasPA grown in the presence of tetracycline, the percentage of cells that kept pCasPA is shown in the last column. (**B**) Growth curves of *B. multivorans* ATCC 17616/pCasPA in Lennox broth medium supplemented with tetracycline 200 µg/mL (*n* = 12) show that the expression of the plasmid-encoded Cas9 induced with L-arabinose up to 2% does not affect growth. The standard deviation of each assay is below 5%.

Plasmid pCasPA expressing *cas9* gene and the genes encoding the proteins of the λ-Red system use the gene conferring resistance to tetracycline as a selection marker. As this antibiotic can select *Burkholderia*-transformed cells, this plasmid was mobilized by triparental conjugation from *E. coli* to *Burkholderia*. For this purpose, we used three strains of *Burkholderia multivorans* (ATCC 17616, P0213-1, and BM1), one of *Burkholderia dolosa* (AU0158), and two of *Burkholderia cenocepacia* (J2315 and K56-2). Plasmid pCasPA was successfully mobilized to all strains, albeit at a different frequency (Fig. 1A). Among *B. multivorans*, the clinical isolates BM1 and P0213-1 showed the highest conjugation frequency [defined by the number of transconjugants divided by the total number of viable cells estimated by colony-forming units (CFU) count and then multiplied by 100]. No colonies were grown in the mating control (absence of the helper strain) implying no spontaneous resistance to tetracycline was observed. *B. dolosa* AU0158 presented an intermediate frequency and *B. cenocepacia* strains displayed the highest conjugation frequency. The assessment of spontaneous tetracycline-resistant colonies in K56-2 resulted in no colonies, but in strain J2315, their presence was at a frequency of 8 × 10^–5^ (data not shown).

Three random colonies of each strain were tested for pCasPA by PCR amplification of an internal fragment of the *cas9* gene. Results shown in Fig. S2 confirmed the *cas9* gene in the tested colonies of all strains but not in the respective wild-type strain used as a negative control. As induction of the λ-Red system and *cas9* gene expression is induced by L-arabinose, we tested the growth of one colony of *B. multivorans* ATCC 17616 harboring pCasPA in the presence of several concentrations of this compound. As shown in Fig. 1B, there is no significant difference in the growth rate of this strain in the absence or presence of arabinose up to 2%, implying that there is no detectable toxicity associated with Cas9. Considering these results, the highest concentration of L-arabinose (2%) was chosen for further experiments.

The next step was the preparation of electrocompetent cells carrying pCasPA subjected to induction of *cas9* and λ-Red system gene expression by 2% L-arabinose. Cells harboring pCasPA were grown in Lennox broth (LB) liquid medium with tetracycline (200 μg/mL for *B. multivorans* and *B. dolosa*, and 400 μg/mL for *B. cenocepacia* J2315 and K56-2) until OD_640_ of 0.8–1. Then, gene expression was induced by L-arabinose for 3 hours, and electrocompetent cells were prepared. We then estimated the percentage of electrocompetent cells that kept pCasPA, with *B. dolosa* AU0158 showing 94% of the cells harboring this plasmid, while the levels of pCasPA in *B. multivorans* ranged from 42% to 69% and *B. cenocepacia* ranged from 13% to 45% (Fig. 1A) showing some instability of this plasmid specially in *B. cenocepacia*.

### Exchange of the antibiotic resistance cassette of pACRISPR

Plasmid pACRISPR encodes the *bla* gene encoding a β-lactamase conferring resistance to ampicillin, carbenicillin, and related antibiotics but is not suitable as a selection marker in *Burkholderia* strains because of their intrinsic resistance to this class of antibiotics. Therefore, the *bla* gene was removed with the restriction enzymes AgeI/BbsI, and genes for three different selection markers were added. These are the gene *cat* encoding a protein conferring resistance against chloramphenicol, the *aph* gene encoding an aminoglycoside 3′-phosphotransferase, conferring resistance against kanamycin, and the *dhfr* gene encoding dihydrofolate reductase conferring resistance against trimethoprim. This gave rise to plasmids pACRISPR-Cm (size of 6,539 bp), pACRISPR-Km (size of 6,711 bp), and pACRISPR-Tp (size of 6,061 bp), conferring resistance to chloramphenicol, kanamycin, and trimethoprim, respectively. None of these gene replacements introduced additional sites for the restriction endonuclease BsaI, which is needed for cloning the spacer, and XbaI and XhoI, which are needed for cloning the homologous repair arms. The three different vectors increase the possibility of at least one of them being possible to use in these highly antibiotic-resistant *Burkholderia* strains. Since the work has been focused on *B. multivorans* strains, only pACRISPR-Km was used.

### Gene deletion in *B. multivorans* P0213-1

**Fig 2:**
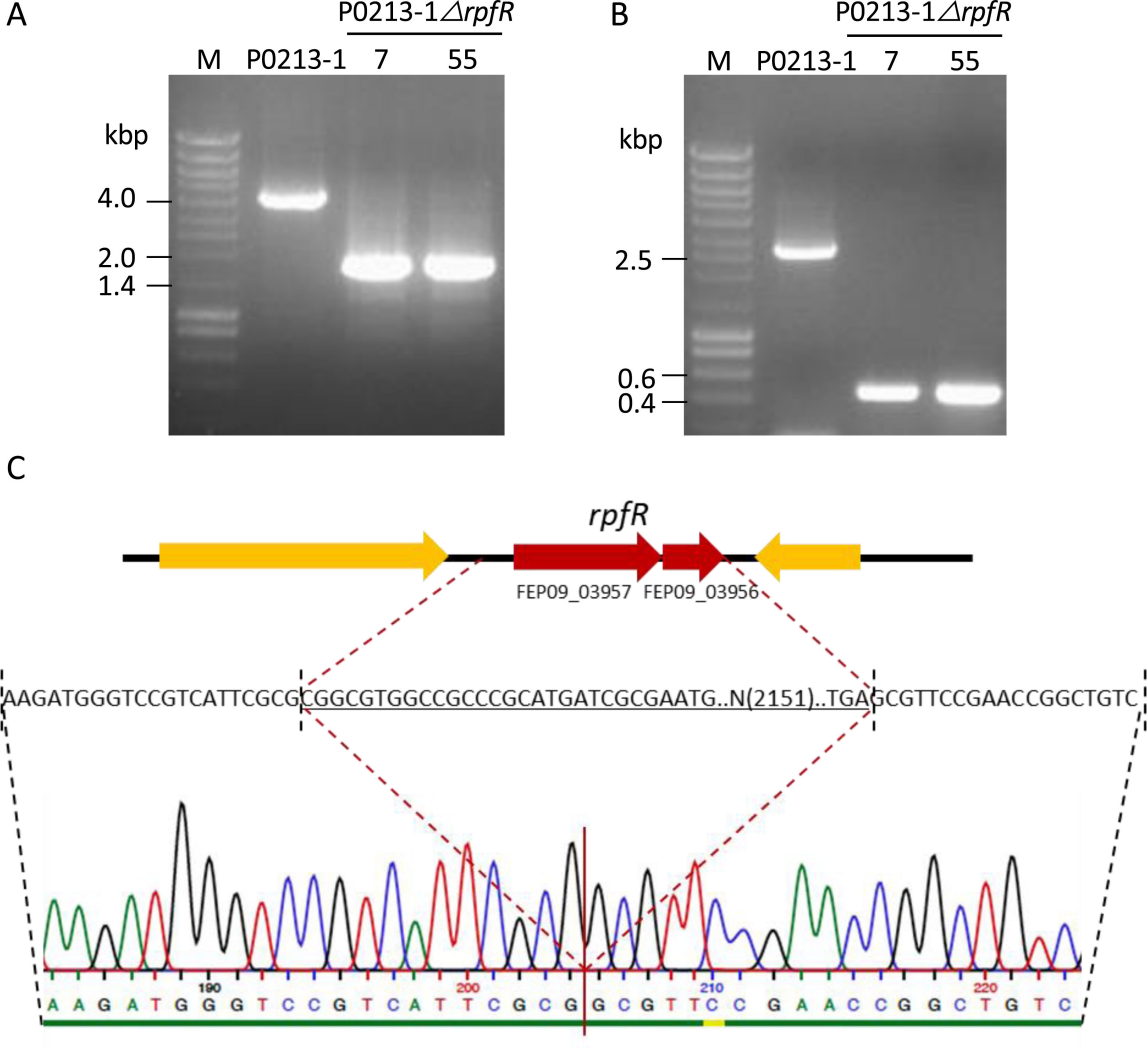
Deletion of *rpfR* coding region from *B. multivorans* P0213-1 genome and mutant genotype confirmation. Electrophoretic separation of the PCR products amplified using two different primer sets from the wild-type strain P0213-1 and two colonies with the deletion of the *rpfR* coding sequences (locus tag: FEP09_03956 and FEP09_03957) before (**A**) and after (**B**) plasmid curing. (**C**) Confirmation of the genotype of the *ΔrpfR* deletion mutant derived from colony 7 by Sanger sequencing. The deleted region is underlined.

To test whether plasmids pCasPA and pACRISPR-Km could be used to delete genes from the clinical isolate P0213-1 genome, we have chosen the *rpfR* gene encoding the receptor of the signaling molecule 2-dodecenoic acid implicated in regulating several virulence traits in *B. cenocepacia* (14). Unlike other *Burkholderia* strains in which RpfR originates from a single gene, the RpfR coding sequence of *B. multivorans* P0213-1 has a premature STOP codon, creating two coding sequences annotated as FEP09_03956 and FEP09_03957. To delete these two genes (collectively named *rpfR*), we designed the spacer and the repair arms, the last ones composed of the region upstream of FEP09_03957 and downstream of FEP09_03956. Cloning of the 20-nucleotide (nt) spacer and repair arms (size between 0.6 and 0.8 kbp each) followed the same procedure as reported by Chen and co-authors (10) and described in the Materials and Methods section. Then, L-arabinose-induced electrocompetent cells of *B. multivorans* P0213-1/pCasPA were transformed with approximately 1 μg of the empty vector pACRISPR-Km, plasmid pACRISPR-Km containing the 20-nt *rpfR* spacer (pMF21-4), and pACRISPR-Km assembled with both spacer and repair arms (pMF21-7) and plated onto LB agar supplemented with 500 μg/mL of kanamycin. As a control, non-induced electrocompetent cells of P0213-1/pCasPA were transformed with pMF21-7. While approximately 80 colonies were obtained by transformation with the empty vector, less than 30 were grown when transformed with the plasmid carrying the 20-nt spacer targeting the *rpfR* gene (data not shown). This result confirms sgRNA efficiency in directing Cas9 endonuclease to the *rpfR* locus and causing a double-stranded break, and consequently a higher cell death rate. Transformation of L-arabinose-induced cells with the plasmid containing the *rpfR* spacer and repair arms resulted in 66 colonies. Each one of these 66 colonies was analyzed by PCR for the amplification of a 3.9-kbp fragment if the *rpfR* gene is present in the genome and 1.7 kbp if it is absent. The efficiency of gene deletion was 2/66. Figure 2A shows the confirmation of the deletion of the *rpfR* gene in the two colonies (numbered 7 and 55). Transformation of non-induced cells with pMF21-7 resulted in 75 colonies, but none were mutant. In another experiment using non-induced P0213-1/pCasPA cells, 1 out of 162 analyzed colonies was a mutant (data not shown), suggesting some basal expression of the *cas9* gene and λ-Red system genes might be sufficient, but with much lower efficiency.

When the 66 colonies were grown in the presence of tetracycline to assess pCasPA presence, approximately 37% were sensitive to this antibiotic, including the two Δ*rpfR* deletion mutant colonies. Although plating the electrotransformed cells in the presence of both antibiotics would have reduced the number of colonies to be analyzed, it would also decrease the chances of finding the deletion mutants in case cells lose pCasPA during electrocompetent cells’ preparation or after cells’ electrotransformation. For this reason, after electroporation of pACRISPR-Km-derivatives containing the spacer and repair arms, transformant cells were selected only for kanamycin resistance.

### Curing of pACRISPR-Km derivative from the *B. multivorans* P0213-1 *ΔrpfR* mutant

As vectors pACRISPR and pCasPA have the *sacB* gene preventing cells from surviving in a medium containing sucrose, we tested whether the *ΔrpfR* mutant harboring the pACRISPR-Km derivative could lose it by plating cells onto LB agar with different concentrations of sucrose (5%, 10%, 15%, and 20%). When the number of CFU was estimated both in LB with/without NaCl and with/without supplementation of different sucrose concentrations, no significant difference was obtained. Similar results were observed when plating cells onto a minimal medium supplemented with 2% mannitol and with/without 5% and 10% of sucrose, implying that sucrose selection cannot be used in *B. multivorans* P0213-1. We then tested growth at a lower temperature (18°C–20°C) with passages to a new liquid LB medium (1:30 dilution) every 24 hours. At each passage, cells were plated on LB agar and approximately 300–500 formed colonies were streaked onto LB agar with and without kanamycin. The absence of growth in plates supplemented with kanamycin showed plasmid loss. Plasmid loss was observed after 24 hours (passage 1) for *ΔrpfR* mutant colony 7 and 48 hours (passage 2) for *ΔrpfR* mutant colony 55. This was further confirmed by PCR amplification of a 458-bp fragment containing the deletion of the *rpfR* gene (Fig. 2B). Genotype confirmation by PCR of the kanamycin-sensitive colonies is mandatory because, during the curing process, mutant cells might have recombined again with the plasmid, resulting in loss of the mutation from the genome. Finally, sequencing of the 458-bp PCR product from these two colonies confirmed gene deletion (Fig. 2C) and the successful construction of *B. multivorans* P0213-1 *ΔrpfR* mutant.

### Deletion of genes *bcsB* and *bceF* from *B. multivorans* P0213-1

**Fig 3:**
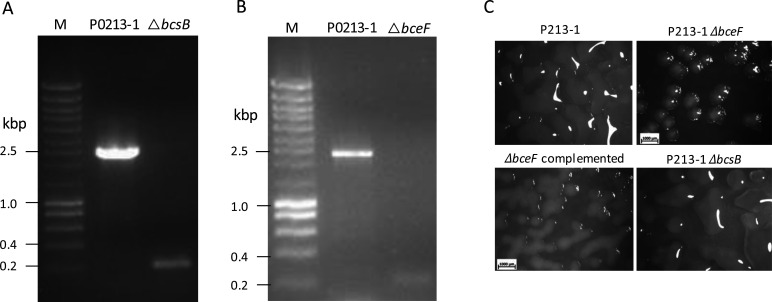
Loss of the *bceF* gene from *B. multivorans* P0213-1 impairs the mucoid phenotype because of cepacian biosynthesis. Confirmation of the deletion of *bcsB* (**A**) and *bceF* (**B**) genes from the P0213-1 strain genome after plasmid curing, as shown by the electrophoretic separation of the amplified PCR products. In the absence of the *bcsB* gene, an amplicon of 223 bp is expected while in the absence of the *bceF* gene, the size is 222 bp. (**C**) Assessment of the mucoid phenotype in the shown strains after growing colonies in yeast extract mannitol medium for 72 hours at 37°C.

After optimization of the procedure to edit the genome of strain P0213-1, we assayed the deletion of two genes within two operons. One of them, *bcsB*, encodes an uncharacterized cellulose synthase regulator protein, possibly implicated in the biosynthesis of a cellulose-like polymer. The other gene, *bceF*, encodes a tyrosine kinase implicated in the biosynthesis of the exopolysaccharide cepacian (15). From the transformation of P0213-1/pCasPA cells with pLM2022-5 containing the *bcsB* spacer and repair arms (size of ~0.9 kbp each), gene deletion occurred with an efficiency of 5/48. Of these five colonies, two of them still harbored the pCasPA plasmid. One of the tetracycline-sensitive *ΔbcsB* mutant colonies was cured from the pACRISPR-Km derivative using the method described above, and the genotype of the mutant is shown in Fig. 3A, with the wild type displaying a band of 2,470 bp and the mutant of 223 bp. To delete the *bceF* gene, plasmid pLM2022-8 containing the *bceF* spacer and repair arms (size between 1 and 1.2 kbp each) was electroporated into P0213-1/pCasPA cells resulting in four deletions in 32 analyzed colonies. None of these colonies had the pCasPA plasmid. One of them was cured from the pACRISPR-Km derivative, and the mutant genotype was confirmed by amplification of a 222-bp fragment in comparison with the 2,412 bp for the wild-type genome (Fig. 3B). The genotypes of mutants *ΔbcsB* and *ΔbceF* confirmed by sequencing are shown in Fig. S3 and S4A, respectively.

Since the *bceF* gene is required for cepacian biosynthesis (15), we compared the mucoid phenotype in the yeast extract mannitol (YEM) medium. As observed in Fig. 3C, the mutant displays a nonmucoid phenotype, while the wild-type produced highly mucoid colonies. The complementation of the *ΔbceF* mutant with a pBBR1MCS-derivative expressing the *bceF* gene in trans resulted in the recovery of the mucoid phenotype. That the *ΔbceF* mutant can be complemented shows that the expression of genes downstream of *bceF* (*bceG* to *bceK*) is not significantly affected by the deletion of this gene. Evaluation of the mucoid trait in the Δ*bcsB* mutant results in similar mucoid levels as the wild-type strain (Fig. 3C), showing that this gene does not contribute to this phenotype.

### Gene deletion in *B. multivorans* ATCC 17616

**Fig 4:**
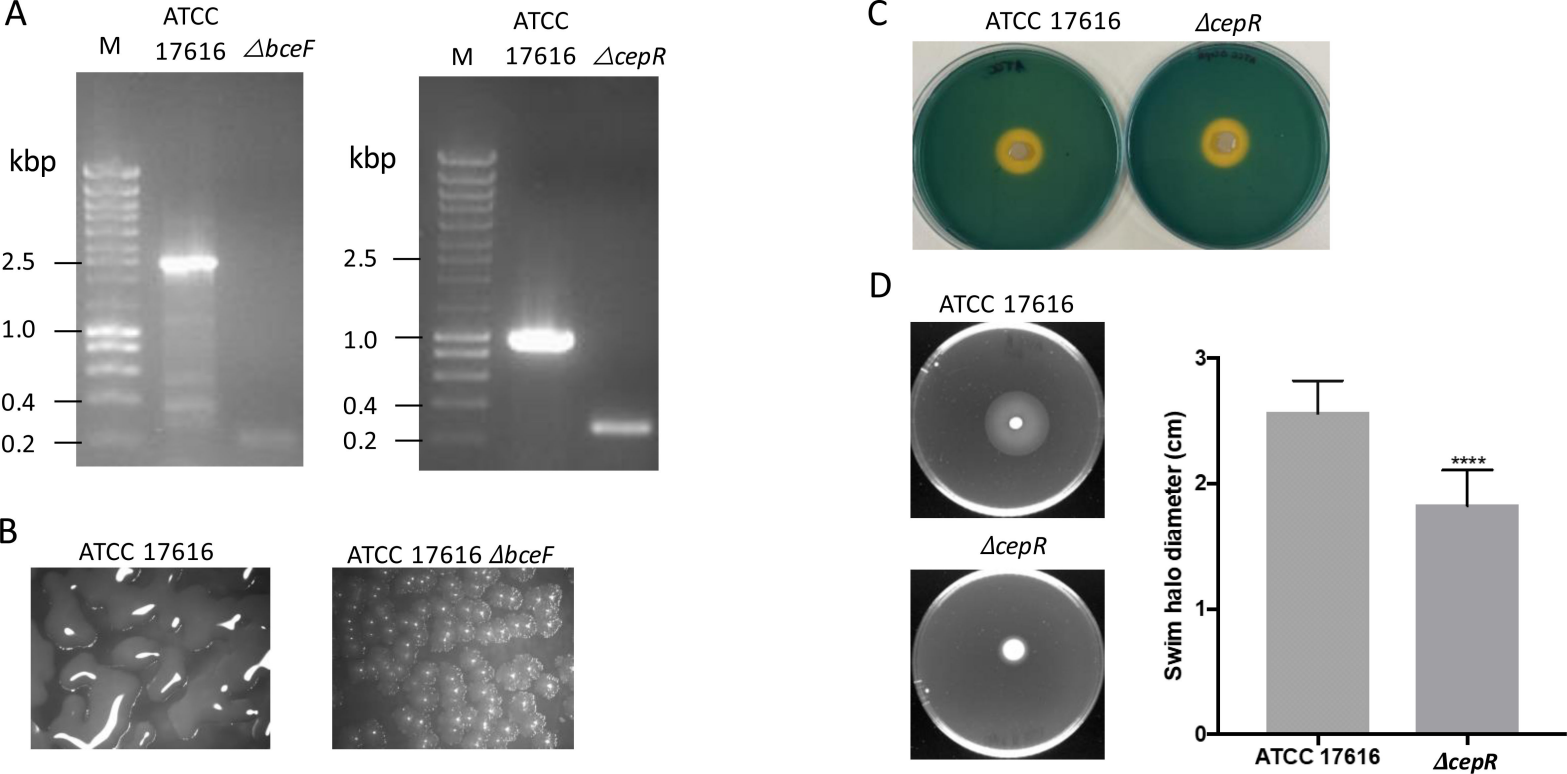
Deletion of the *cepR* gene of *B. multivorans* ATCC 17616 impairs swimming motility but not siderophore biosynthesis. (**A**) Electrophoretic separation of PCR amplicons confirms the absence of the *bceF* gene (expected size 222 bp) or the *cepR* gene (expected size 270 bp) from the ATCC 17616 genome. (**B**) Assessment of the mucoid phenotype in the wild-type and *ΔbceF* mutant after growth at 37°C for 72 hours. (**C**) Siderophore production in MM-CAS medium after 72 hours at 37°C. (**D**) Swimming motility of the wild-type ATCC 17616 and *ΔcepR* mutant (images on the left) and its quantification (*n* = 12). Swimming plates were incubated for 24 hours at 37°C. The *ΔcepR* mutant differed significantly from the wild-type strain for swimming motility. ***P < 0.001 by Tukey’s honestly significant difference multiple-comparison test.

To test whether the CRISPR/Cas9 system under investigation could delete genes from the genome of another *B. multivorans* strain, namely the ATCC 17616, we have also chosen the *bceF* gene and the *cepR* gene encoding the acyl homoserine lactone-based quorum-sensing regulator implicated in the expression of several virulence traits in *Burkholderia* (16). After preparation of plasmids with the spacer and repair arms (pLM2022-9 for *bceF* gene deletion and pVQ2022-2 for *cepR* gene deletion), these were electroporated into strain ATCC 17616/pCasPA. The number of kanamycin-resistant colonies obtained in each electroporation was below 50, confirming previous observations in strain P0213-1, where a reduced number of colonies were obtained. Genotype analysis confirmed gene deletion with an efficiency of 3/40 for the *bceF* gene and 2/40 for the *cepR* gene. These five mutant colonies were resistant to kanamycin and tetracycline because of the presence of pACRISPR-Km derivative and pCasPA, respectively. For plasmid curing, one colony of each mutant was selected and grown at 20°C, with a passage every 24 hours. Our first selection was for the absence of the pACRISPR-Km derivative, which was attained after passage 1 for each of the mutants. Then, these cured colonies, which still harbored pCasPA, were grown at 20°C, and only after passage 3 were the mutants cured from pCasPA. The genotype of both deletion mutants analyzed by PCR amplification of the region flanking the deletion is shown in Fig. 4A and confirmation by DNA sequencing in Fig. S4B and S5A.

To confirm the absence of the mucoid phenotype in the *B. multivorans* ATCC *ΔbceF*, cells were grown in the YEM medium for 72 hours. As shown in Fig. 4B, the wild type is mucoid, while the mutant colonies are nonmucoid, confirming previous observations in the P0213-1 strain. Siderophore production and motility are two phenotypes known to depend on quorum-sensing regulation in *B. cenocepacia* (16, 17). To evaluate whether the deletion of the *cepR* gene would affect these phenotypes in *B. multivorans* ATCC 17616, we assessed both the wild-type and mutant strains. Growing cells in MM-CAS medium confirmed the biosynthesis of siderophore by the wild-type ATCC 17616 but unexpectedly also by the *ΔcepR* mutant (Fig. 4C), suggesting that regulation of gene expression toward siderophore biosynthesis in this strain differs from *B. cenocepacia* K56-2. In contrast, motility assays showed the reduced ability of the *ΔcepR* mutant to swim, as stated in Fig. 4D. The *ΔcepR* mutant displayed a halo diameter of 1.8 cm, while the wild-type strain halo diameter had 2.6 cm. Similar results of siderophore production and motility were obtained for a *B. multivorans* P0213-1 *ΔcepR* mutant (data not shown).

### Gene insertion in *B. multivorans* ATCC 17616

**Fig 5:**
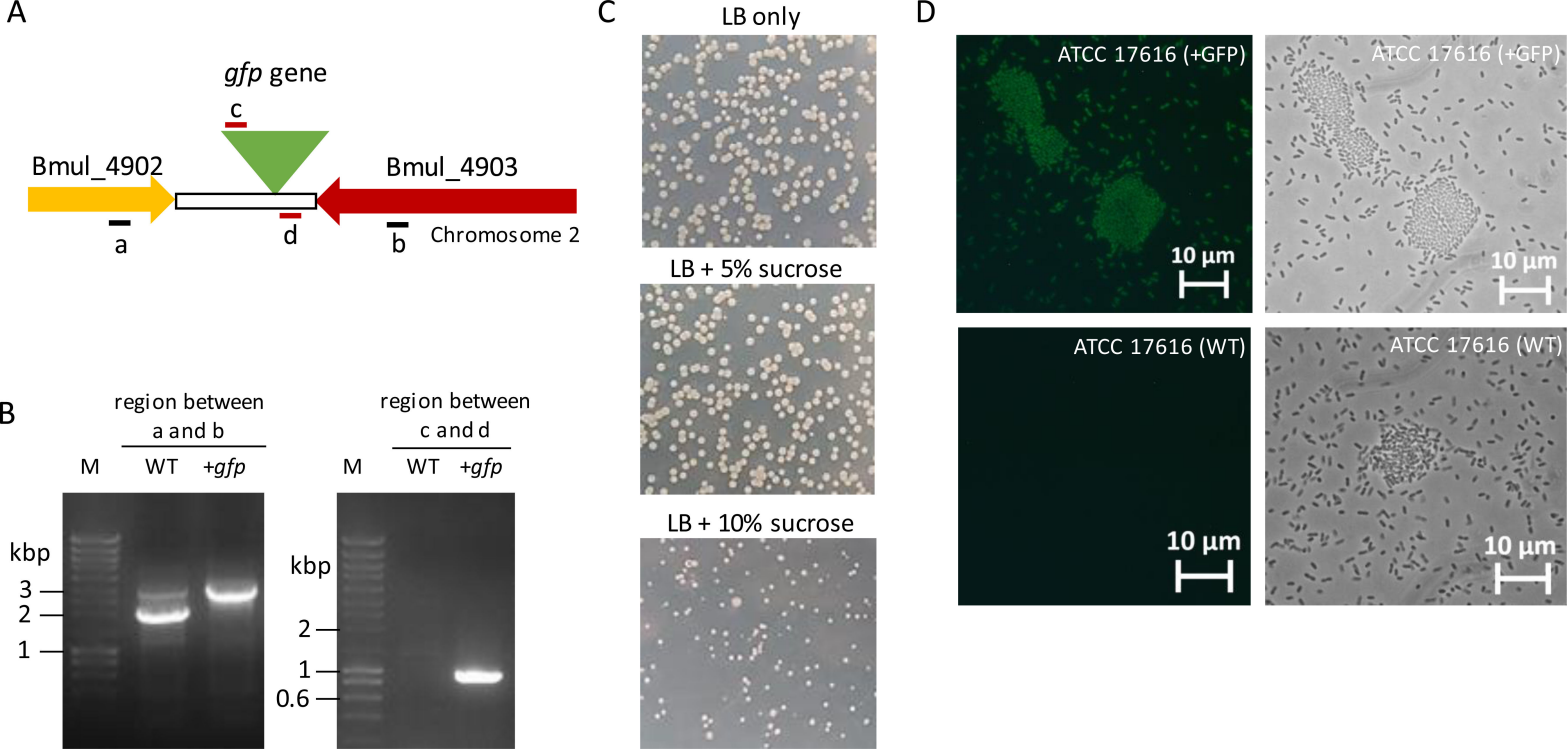
Gene *gfp* was successfully inserted into the genome of *B. multivorans* ATCC 17616. (**A**) Schematic representation of the intergenic region between Bmul_4902 and Bmul_4903 highlighting the place of *gfp* gene insertion (showed by a green triangle). Letters a/b and c/d show the position of the primers selected to confirm the insertion of the *gfp* gene before and after plasmid curing, respectively. (**B**) Electrophoretic separation of the PCR products confirming *gfp* gene insertion into the genome tested with primers for regions a/b (left panel) and c/d (right panel), this last after plasmid curing. (**C**) Curing of pCasPA and pACRISPR-Km derivatives using the activity of the *sacB* gene product. Cells were plated in the shown medium and incubated at 37°C for 24 hours. (**D**) Fluorescence microscopy (left panel) and light microscopy (right panel) images showing cells of wild-type ATCC 17616 and ATCC 17616 with the gene *gfp* inserted into chromosome 2.

To test whether the CRISPR/Cas9 system could insert DNA fragments into the *B. multivorans* ATCC 17616 genome at precise locations, we selected the *gfp* gene encoding the green fluorescence protein. The selected site of insertion was in chromosome 2 and comprised an intergenic region of 813 bp between the end of the coding sequence of two genes (Bmul_4902 and Bmul_4903) convergently transcribed (Fig. 5A). The repair arms to be used were the region upstream (709 bp) and downstream (770 bp) of the region where Cas9 should cut, with the *gfp* gene and promoter amplified from pIN25 vector, being inserted between the two regions. This plasmid, pVQ2022-1, was electroporated into *B. multivorans* ATCC 17616/pCasPA, resulting in 12 colonies. After genotype analysis, 2 out of 12 colonies had the *gfp* gene inserted into the genome (Fig. 5B, left image). Colony 1 was already cured from pCasPA, while colony 2 still had both plasmids. Besides the established plasmid curing method for P0213-1 and ATCC 17616 described above, here we tested curing in the presence of sucrose since this is a different *B. multivorans* strain. For that, the ATCC 17616::*gfp* colonies 1 and 2 were plated in LB agar only or supplemented with 5% and 10% of sucrose. After 24 hours of incubation, the number of CFU in the presence of 5% sucrose was like LB agar only (Fig. 5C), but when in the presence of 10% sucrose, the number of CFU was considerably lower and colonies were smaller, suggesting that this concentration had some negative effect on cell viability. Several of these colonies were tested for kanamycin sensitivity and then selected for genotype confirmation by PCR. Sixteen cured colonies from ATCC 17616::*gfp* colony 1 (tetracycline and kanamycin sensitive) analyzed for *gfp* gene insertion confirmed its presence in nine of them, with the remaining having lost the insertion and becoming wild type again (data not shown). From ATCC 17616::*gfp* colony 2, we selected eight kanamycin-sensitive colonies, with two of them having the *gfp* gene inserted into the genome. These colonies were still tetracycline resistant and would need another round of curing. Confirmation of the ATCC 17616::*gfp* genotype is shown in Fig. 5B (right image), where the *gfp* gene amplified while no amplification was obtained in the wild-type strain, shown in Fig. S6. Fluorescence microscopy images confirm the fluorescence of cells of ATCC 17616::*gfp* producing GFP protein and the absence of signal in ATCC 17616 (Fig. 5D).

## DISCUSSION

Genetic tools to introduce changes into bacterial genomes are crucial to understanding the processes governing cell behavior in different environments. In this study, we successfully optimized a CRISPR/Cas9 system for efficient editing of *B. multivorans* genomes, with the key steps shown in Fig. S7. By exchanging the antibiotic selection marker of pACRISPR vector, optimizing gene expression induction, and plasmid curing, we could genetically manipulate the genome of *B. multivorans* strains in a precise and seamless manner. The process is as efficient for gene deletion as for gene insertion, and the simplified cloning of the spacer and repair arms and enhanced homologous recombination activity because of the λ-Red system expression in *B. multivorans* make this method an excellent option for manipulating the genome of these bacteria.

One major question was whether Cas9 production from plasmid pCasPA would cause toxicity, as previously reported for other bacteria, such as *E. coli*, *Clostridium butyricum*, and *Mycobacterium* (18–21). Our experiment showing that different concentrations of the inducer L-arabinose do not negatively impact cell growth suggests that no significant proteotoxicity must be present. Yet, we observed instability of pCasPA, with plasmid loss being significant even in the presence of selective pressure applied to cell cultures when competent cells are being prepared. Although pCasPA loss certainly has a negative impact on the efficiency of genome editing, it is also possible that if this loss occurs after the induction of the *araB* promoter, there are sufficient levels of Cas9 and λ-Red system proteins for this method to work. Effectively, *B. multivorans* mutants that had lost pCasPA were often identified.

Studies in other bacterial systems showed that the λ-Red system proteins need to be expressed before the CRISPR/Cas9 activation for maximal double-strand break recovery through homologous recombination (22). Our work corroborates these results, as editing frequency was higher in L-arabinose-induced cells, but even without induction, the basal expression of genes from pCasPA still allowed the recovery of mutant colonies, despite at a much lower frequency. Another crucial step is the design of spacer regions to direct Cas9 to the targeted locus. Although different spacers might have very different efficiencies, our criterion of choosing a match either to the forward or reverse strands at the middle of the gene-coding sequence to be deleted was always successful. Our data showed efficient sgRNA results in a low number of transformed colonies (even if the repair arms are present), as expected from the lethality of the double-strand break because of Cas9 activity and the low efficiency of the endogenous repair systems. Regarding the repair template, although the pCasPA/pACRISPR system of *Pseudomonas* was successful in providing it in circular and linear double-strand forms in sizes ranging from 100 to 1,000 bp (10), the ones presenting higher efficiency of editing included 500–1,000 bp in size and were provided in a plasmid. Therefore, we only tested repair arms of sizes ranging from 600 to 1,200 bp cloned into pACRISPR derivatives. Because of the size of the regions needed for homologous recombination and the easy method to assemble them into a circular form, associated with a slightly lower efficiency using linear fragments as described by Chen and co-authors (10), this is proved to be an adequate strategy.

After the identification of mutants with the edited genome, it was necessary to cure the plasmids to have a stable mutation. Despite the *sacB* gene being present in both plasmids, the sucrose-mediated selection of the cured colonies could only be used in *B. multivorans* ATCC 17616 but not in the P0213-1 strain. In the literature, other methods for curing plasmids, such as growing under nutrient limitation, high or low temperature of growth, and mutagenic compounds, among others can be found (23–25). We have tested some of these methods and were only successful when growing P0213-1 mutants with pACRISPR-Km derivatives and/or pCasPA under a sub-optimal temperature of 18°C–20°C. Under these conditions, pCasPA was easily lost, but the pACRISPR-Km derivative could require more passages and, therefore, a longer time until the cured mutant was identified. Sucrose-mediated selection of pACRISPR cured colonies was effective in *B. multivorans* ATCC 17616, but the colonies still had the pCasPA plasmid. This can be explained by the lower expression of *sacB* gene from this single copy number plasmid. Nevertheless, the growth of mutant cells at lower temperatures also induces pCasPA loss, and a combination of the two methods can achieve a loss of both plasmids.

A comparison of the methods developed by Flannagan and co-authors (5,6) and by Fazli and co-authors (6,6) to mutagenize *Burkholderia* genomes shows differences in the way the flanking regions of the gene to be deleted are cloned and on the final plasmid curing method, but both use a suicide vector containing the I-SceI endonuclease restriction site, which carries the flanking regions of the gene to be deleted. This plasmid integrates into the genome forming merodiploids, which need to be resolved by another recombination event induced by the cutting of the I-SceI endonuclease, expressed from a second replicative plasmid. Compared to these two steps needed to replace the wild-type copy of the gene by the mutant copy, our system has the advantage of needing just one step of recombination for the exchange of the wild-type gene by the mutant copy, since no plasmid integration in the genome is required. Furthermore, the cloning into pACRISPR vector of the spacer by Golden Gate and of the homologous repair arms by Gibson assembly are easier than the large amplification products needed in the second step of Gateway cloning, especially if the system would be used for gene insertion instead of gene deletion. Independent of these differences, our work adds another tool to manipulate the genome of these microorganisms.

Compared with other CRISPR/Cas systems already used in *Burkholderia*, our system is optimized to address specific limitations found in these other systems. While the CRISPR-associated transposase system has a high efficiency in disrupting genes in *Burkholderia thailandensis*, it is not suitable for the construction of deletion mutants, and transposon integration problems may extend the time needed to obtain the desired mutants (7). The system used by Deng and co-authors (9), expressing the Cas9, an editing template, and a targeting construct, could only generate small site-specific deletions in a *B. contaminans* MS14 gene typical of the non-homologous end-joining double-strand break repair system. Here, disruption of gene activity was attained, but the system was not tested for larger deletions or gene insertion. Moreover, there is no information on the efficiency of this system.

This promising genetic tool, here adjusted for *B. multivorans,* can be a starting point to develop a similar approach for other *Burkholderia* species. Effectively, we have shown that pCasPA can be stably transformed into *B. cenocepacia* and *B. dolosa*, suggesting no toxicity by the *cas9* gene expression, but additional studies are needed. Altogether, the genome editing method here optimized allows for fast and cost-effective genomic manipulation in *B. multivorans*, contributing to accelerating research in these under-studied microorganisms of broad environmental and clinical importance.

## MATERIALS AND METHODS

### Bacterial strains, plasmids, and growth conditions

Bacterial strains and plasmids used in this work are listed in Table 1. *Escherichia coli* and *Burkholderia* strains were routinely grown at 37°C in Lennox broth with or without agar supplemented with antibiotics (Sigma-Aldrich) when required. Antibiotic concentration for *E. coli* was tetracycline, 10 μg/mL; kanamycin, 50 μg/mL; chloramphenicol, 25 μg/mL; and trimethoprim, 100 μg/mL. Antibiotic concentration for *B. multivorans* was tetracycline, 200 μg/mL; trimethoprim, 300 μg/mL; chloramphenicol, 200 μg/mL; and kanamycin, 500 μg/mL. For *B. cenocepacia* and *B. dolosa*, 300 and 200 μg/mL, respectively, of tetracycline was used. For phenotypic tests, *B. multivorans* strains were grown in an LB or salts-mannitol (SM) medium (26).

**TABLE 1:** Bacterial strains and plasmids^*a*^

Strains/plasmids	Description	Reference/source
Bacterial strains		
*Burkholderia multivorans* ATCC 17616	Soil isolate, USA	(27)
*Burkholderia multivorans* P0213-1	Cystic fibrosis isolate VC7495, Canada	CBCCRRR
*Burkholderia multivorans* BM1	Cystic fibrosis isolate, Canada	(28)
*Burkholderia dolosa* AU0158	Cystic fibrosis isolate, USA	CBCCRRR
*Burkholderia cenocepacia* J2315	Cystic fibrosis isolate, ET12 index strain, Edinburgh, UK	(29)
*Burkholderia cenocepacia* K56-2	Cystic fibrosis isolate, ET12, Toronto, Canada	(30)
*B. multivorans* P0213-1 *ΔrpfR*	P0213-1 with genes FEP09_03956 and FEP09_03957 (*rpfR*) deleted	This work
*B. multivorans* P0213-1 *ΔbcsB*	P0213-1 with gene FEP09_02916 (*bcsB*) deleted	This work
*B. multivorans* P0213-1 *ΔbceF*	P0213-1 with gene FEP09_03744 (*bceF*) deleted	This work
*B. multivorans* P0213-1 *ΔcepR*	P0213-1 with gene FEP09_04935 (*cepR*) deleted	This work
*B. multivorans* ATCC 17616 *ΔbceF*	ATCC 17616 with gene Bmul_4915 (*bceF*) deleted	This work
*B. multivorans* ATCC 17616 *ΔcepR*	ATCC 17616 with gene Bmul_3971 (*cepR*) deleted	This work
*B. multivorans* ATCC 17616::*gfp*	ATCC 17616 with gene *gfp* inserted in the intergenic region between Bmul_4902 and Bmul_4903	This work
*Escherichia coli* DH5α	Δ(*lacZYA-argF*)*U169 ф*80*dlacZ*ΔM15*recA1*	Gibco BRL
Plasmids		
pCasPA	Bacterial expression of Cas9 nuclease and λ-Red recombination system; Tc^R^	(10)
pACRISPR	Plasmid for the expression of sgRNA for targeting specific bacterial sequences and cloning of homologous arms; Ap^R^	(10)
pACRISPR-Km	pACRISPR derivative with the *bla* gene replaced by the *aph* gene; Km^R^	This work
pACRISPR-Cm	pACRISPR derivative with the *bla* gene replaced by the *cat* gene; Cm^R^	This work
pACRISPR-Tp	pACRISPR derivative with the *bla* gene replaced by the *dhfr* gene; Tp^R^	This work
pLM127-13	pBBR1MCS derivative expressing the *bceF* gene of *B. contaminans* IST408 under the control of the *bce* operon promoter; Cm^R^	(31)
pK19mob	pUC18/19 derivative, *lacZα*, *mob*, Km^R^	(32)
pBBR1MCS	4,717-bp broad host range cloning vector, Cm^R^	(33)
pUC-TP	pUC-GM derivative with a 1.1 kb Tp^R^ gene cassette, Ap^R^, Tp^R^	(34)
pRK2013	Tra^+^ Mob^+^ (RK2) Km::Tn7 ColE1 origin, helper plasmid in triparental mating, Km^R^	(35)
pRK600	ColE1oriV, RK-2Mob^+^ RK2-Tra^+^, helper plasmid in triparental mating, Cm^R^	(36)
pIN25	*ori*_pBBR_, Δ*mob*, GFP; Cm^R^	(37)
pMF21-4	pACRISPR-Km derivative containing the 20-nt spacer targeting *B. multivorans* P0213-1 *rpfR* gene; Km^R^	This work
pMF21-7	pMF21-4 derivative containing the upstream 779 bp and the downstream 620 bp regions of the *rpfR* gene; Km^R^	This work
pLM2022-4	pACRISPR-Km derivative containing the 20-nt spacer targeting *B. multivorans* P0213-1 *bcsB* gene; Km^R^	This work
pLM2022-5	pLM2022-4 derivative containing the 940 bp region upstream and the 912 bp region downstream of the *bcsB* gene; Km^R^	This work
pLM2022-6	pACRISPR-Km derivative containing the 20-nt spacer targeting *B. multivorans* P0213-1 *bceF* gene; Km^R^	This work
pLM2022-7	pACRISPR-Km derivative containing the 20-nt spacer targeting *B. multivorans* ATCC 17616 *bceF* gene; Km^R^	This work
pLM2022-8	pLM2022-6 derivative containing the 971 bp region upstream and the 1,189 bp region downstream of *bceF* gene from *B. multivorans* P0213-1; Km^R^	This work
pLM2022-9	pLM2022-7 derivative containing the 971 bp region upstream and the 1,189 bp region downstream of *bceF* gene from *B. multivorans* ATCC 17616; Km^R^	This work
pLM2022-11	pACRISPR-Km derivative containing the 20-nt spacer targeting *B. multivorans* ATCC 17616 *cepR* gene; Km^R^	This work
pVQ2022-2	pLM2022-11 derivative containing the 939 bp region upstream and the 936 bp region downstream of the *cepR* gene from *B. multivorans* ATCC 17616; Km^R^	This work
pLM2022-12	pACRISPR-Km derivative containing the 20-nt spacer targeting *B. multivorans* P0213-1 *cepR* gene; Km^R^	This work
pVQ2022-3	pLM2022-12 derivative containing the 939 bp region upstream and the 936 bp region downstream of the *cepR* gene from *B. multivorans* P0213-1; Km^R^	This work
pLM2022-10	pACRISPR-Km derivative containing the 20-nt spacer targeting *B. multivorans* ATCC 17616 intergenic region (IG) between Bmul_4902 and Bmul_4903 genes; Km^R^	This work
pVQ2022-1	pLM2022-10 derivative containing the 709 bp IG region upstream of the Cas9 cutting site, the 797 bp *gfp* gene from pIN25, and the 770 bp IG region downstream of the Cas9 cutting site, from *B. multivorans* ATCC 17616; Km^R^	This work

^*a*^ Km^R^, kanamycin resistance; Cm^R^, chloramphenicol resistance; Tp^R^, trimethoprim resistance; Tc^R^, tetracycline resistance; and Ap^R^, ampicillin resistance. CBCCRRR, Canadian *Burkholderia cepacia* complex research and referral repository.

### DNA manipulation techniques

Total DNA isolation was performed by using the DNeasy Blood & Tissue kit (Qiagen) according to the manufacturer’s instructions. When colony PCR was done, cells were boiled for 5 minutes, followed by centrifugation to recover the total DNA from the supernatant. Plasmid DNA isolation, DNA restriction, agarose gel electrophoresis, and DNA amplification by PCR were performed using standard procedures (38). The kit NZYMiniprep (NZYtech) was used for cloning vector extraction and purification. PCR product purification was performed with the DNA clean & concentrator kit (Zymo Research).

### Modification of pACRISPR vector

To replace the antibiotic selection marker of pACRISPR, this vector was digested with AgeI and BbsI restriction endonucleases, releasing the *bla* gene. Then, the *aph* gene encoding resistance to kanamycin, the *cat* gene encoding resistance to chloramphenicol, and the *dhfr* gene encoding resistance to trimethoprim were amplified by PCR using pK19mob, pBBR1MCS, and pUC-TP vectors, respectively, as templates (primers are indicated in Table 2). The PCR products were digested with AgeI and BbsI and cloned into pACRISPR digested with the same enzymes. The new vectors named pACRISPR-Km, pACRISPR-Cm, and pACRISPR-Tp (deposited at Addgene) were confirmed by sequencing the region containing the antibiotic selection marker.

**TABLE 2:** List of primers used in this work

Name	Sequence 5′−3′	Description
Confirm pCasPA in *Burkholderia*		
confCas9_F confCas9_R	GGAAGCGACTCGTCTCAAAC TTCTTCTTGGCTAGCTCCCC	Amplification of a 937-bp internal fragment from the beginning of *cas9* gene present in plasmid pCasPA Amplification of a 936-bp internal fragment from the end of *cas9* gene present in plasmid pCasPA
confCas9_2_F confCas9_2_R	AGCTGAACGTGGAGGTTTGA AGCATCCGTTTACGACCGTT	
Exchange of selection marker in pACRISPR		
cat_bbsI_F	C*GAAGAC*GAAAGGTCGAATAAATACCTGTGACGGA	Amplification of *cat* coding sequence and promoter region from pBBR1MCS
cat_ageI_R	CC*ACCGGT*GCTGCATTAATGAATCGGCC	
Km_pACRISPR_F	*GAAGAC*GAAAGGATGTCAGCTACTGGGCTATC	Amplification of aminoglycoside 3′-phosphotransferase coding sequence and promoter region from pK19mob
Km_pACRISPR_R	*ACCGGT*GTCATTTCGAACCCCAGAGTC	
dhfr_bbsI_F	*GAAGAC*GAAAGGCCGTGGGTCGATGTTTGATG	Amplification of *dhfr* coding sequence and promoter region from pUC-TP
dhfr_ageI_R	*ACCGGT*CTGGAGCGAATTGTTAGGCC	
Test spacer presence		
test_spacer_F	GGATTTGCAGACTACGGGCCTA	Amplification of the region where spacer insertion occurs
test_spacer_R	CGACTCGGTGCCACTTTTTCAA	
Deletion of the *rpfR* gene in P0213-1		
rpfR_spacer_F	GTGGGGTATCGGTGTACGCCGACG	*rpfR* spacer for gene deletion in P0213-1
rpfR_spacer_R	AAACCGTCGGCGTACACCGATACC	
rpfR_P1	TTTTGAGATCTGTCCATACCCATGG*TCTAGA*TGCAGACGCACATGACGAA	Amplification of 779 bp region upstream *rpfR*
rpfR_P2	GACAGCCGGTTCGGAACGCCGCGAATGACGGACCCATCTT	
rpfR_P3	AAGATGGGTCCGTCATTCGCGGCGTTCCGAACCGGCTGTC	Amplification of 620 bp region downstream *rpfR*
rpfR_P4	TCTGAATGGCGGGAGTATGAAAAGT*CTCGAG*GGCGCGATCTGCTGATGGCAT	
conf_rpfR_F	CAGGGGACGATGAACAACTT	Amplification of 1.663 bp to confirm the mutant (3.846 bp in the wild type)
conf_rpfR_R	CTTCAACCAGCAACTCGTCA	
conf_rpfR_final_F	AGCATTCCGTCCCCAAAACGC	Amplification of 458 bp to confirm *rpfR* deletion (2,641 bp in the wild type)
conf_rpfR_final_R	AGCCGAAGCTGAACGGGATTC	
Deletion of the *bcsB* gene in P0213-1		
bcsB_spacer_F	GTGGTGCAGGACAATTCGACGCTC	*bcsB* spacer for gene deletion in P0213-1
bcsB _spacer_R	AAACGAGCGTCGAATTGTCCTGCA	
bcsB _P1	TTTTGAGATCTGTCCATACCCATGG*TCTAGA*TTGAACGTGTCGGTGATCTG	Amplification of 940 bp region upstream *bcsB*
bcsB _P2	CACCATCCGTTACATCCCCGCAGCTGCATGGAATGTG	
bcsB _P3	CACATTCCATGCAGCTGCGGGGATGTAACGGATGGTG	Amplification of 912 bp region downstream *bcsB*
bcsB _P4	TCTGAATGGCGGGAGTATGAAAAGT*CTCGAG*ATGCCGACCCACAGATAGAC	
conf_ bcsB_CR_F	GTTTTCGTTCGTGGAGGCAT	Amplification of 2,028 bp to confirm the mutant (4.275 bp in the wild type)
conf_ bcsB_CR_R	TCGTGTCGACTTTCTCCGG	
conf_bcsB _final_F	CGCCGTTTCGATAAATGAAT	Amplification of 223 bp to confirm *bcsB* deletion (2,470 bp in the wild type)
conf_bcsB _final_R	CCGCACCATCCGTTACAT	
Deletion of the *bceF* gene in P0213-1 and ATCC 17616		
sg_bceF_ATCC_F	GTGGTCCGTCGGAGTTGCTGATGT	*bceF* spacer for gene deletion in ATCC 17616
sg_bceF_ATCC_R	AAACACATCAGCAACTCCGACGGA	
sg_bceF_F	GTGGCAACATCCATCTGATCGATT	*bceF* spacer for gene deletion in P0213-1
sg_bceF_R	AAACAATCGATCAGATGGATGTTG	
bceF_P1	TTTTGAGATCTGTCCATACCCATGG*TCTAGA*GATCACCCGGAGCTGACG	Amplification of 971 bp region upstream *bceF*
bceF_P2	CATGCGCTCAGGTATTCGTGTTGCGTGTTCACCATTCGTT	
bceF_P3	AACGAATGGTGAACACGCAACACGAATACCTGAGCGCATG	Amplification of 1,189 bp region downstream *bceF*
bceF_P4	TCTGAATGGCGGGAGTATGAAAAGT*CTCGAG*CGTGGTGATAGCCGTACAGA	
conf_bceF_CR_F	GTGTATCACGTCGGCCCG	Amplification of 2,222 bp to confirm the mutant (4,412 bp in the wild type)
conf_bceF_CR_R	GAATTCGTCCGATTCCGCG	
conf_bceF_F	CTTCTACATGCGGCAGATCA	Amplification of 222 bp to confirm the mutant (2,412 bp in the wild type)
conf_bceF_R	GGTAGGTCGGAACGAGCAC	
Deletion of the *cepR* gene in P0213-1 and ATCC 17616		
sg_cepR_P0213-1_F	GTGGCCATGCCGATCGGCTCACAC	cepR spacer for gene deletion in P0213-1
sg_cepR_P0213-1_R	AAACGTGTGAGCCGATCGGCATGG	
sg_cepR_F	GTGGACGGCTGGATGGCGCACTAT	*cepR* spacer for gene deletion in ATCC 17616
sg_ cepR _R	AAACATAGTGCGCCATCCAGCCGT	
cepR _P1	TTTTGAGATCTGTCCATACCCATGG*TCTAGA*CACAACCGCACATCTCGC	Amplification of 939 bp region upstream *cepR*
cepR _P2	AGGGCGTATCGATGAGTCCCCAGCGCAGTTCCATTCTTT	
cepR _P3	AAAGAATGGAACTGCGCTGGGGACTCATCGATACGCCCT	Amplification of 936 bp region downstream *cepR*
cepR _P4	TCTGAATGGCGGGAGTATGAAAAGT*CTCGAG*CGCCTACCGTTTCACACTG	
conf_ cepR _F	AACAGTCGTTCCATGCTCG	Amplification of 2,225 bp to confirm the mutant (2,909 bp in the wild type)
conf_ cepR _R	GTGGACAGTTGCAGCACTC	
conf_ cepR_final_F	TTTCTGACAGGCGCACATAG	Amplification of 270 bp to confirm the mutant (954 bp in the wild type)
conf_ cepR_final_R	GGAGCCGATGATGGAGTGA	
Insertion of *gfp* gene in ATCC 17616		
sg_IG_F	GTGGAAGGTTGCGGATCTGAAACA	IG region spacer for *gfp* gene insertion in ATCC 17616
sg_IG_R	AAACTGTTTCAGATCCGCAACCTT	
IG_P1	TTTTGAGATCTGTCCATACCCATGG*TCTAGA*CTTTCTGAAGTCGCCGCG	Amplification of 709 bp region upstream of the Cas9 cutting site
IG_P2	TGATTAATTGTCAACAGCTCCGATGGGCATGCGAAAGTC	
IG_P3	GACTTTCGCATGCCCATCGGAGCTGTTGACAATTAATCA	Amplification of a 797 bp fragment containing the *gfp* coding sequence and promoter
IG_P4	CGATGGGCATGCGAAAGTCCTATTTGTATAGTTCATCCA	
IG_P5	TGGATGAACTATACAAATAGGACTTTCGCATGCCCATCG	Amplification of 770 bp region downstream of the Cas9 cutting site
IG_P6	TCTGAATGGCGGGAGTATGAAAAGT*CTCGAG*CAGAAGTACAAGGCCGACAG	
Conf_gfp_F	GAATCGCTGAAGGGCAAGTC	Amplification of 2,276 bp to confirm *gfp* gene insertion (1,922 bp in the wild type)
Conf_gfp_R	CACGCTGTTTCTCGACGAA	
Conf_gfp_final_F	CCATCGGAGCTGTTGACAAT	Amplification of 936 bp to confirm *gfp* gene insertion (no amplification in the wild type)
Conf_gfp_final_R	GGACCATGTTTCAGATCCGC	

^*a*^ Restriction sites are in italic and underlined.

### Spacer design and construction of gRNA-expressing plasmids

Spacer design was carried out with the web tool CRISPOR (39) against the genome of each *Burkholderia multivorans* strain under study and 20 bp-NGG protospacer adjacent motif selection. From the identified spacers, we selected the ones with zero off-targets and then ranked them by the predicted efficiency and cutting position within the coding sequence of a gene (preferably in the middle region). The best spacer sequence was selected and then, to the 5′ end of the forward and reverse oligonucleotides, nucleotides compatible with the BsaI restriction endonuclease were added (spacer sequences are in Table 2). Cloning the spacer into pACRISPR-Km followed the procedure described previously (10). Briefly, each pair of forward and reverse oligonucleotides (1 μL of a stock of 50 μM) was phosphorylated by T4 polynucleotide kinase for 1 hour at 37°C. For the annealing, 2.5 μL of NaCl was added, followed by incubation at 95°C for 3 minutes, and then slowly cooled down to room temperature using a thermocycler. The annealed oligos, diluted 20-fold, were added to the Golden Gate assembly mix to insert the spacer into the BsaI site of pACRISPR-Km, as per the instructions from New England Biolabs. After purification, the Golden Gate assembly product was electroporated into *E. coli* cells. The success of pACRISPR-Km_spacer construction was verified by PCR amplification (primers Test_spacer_F/R, Table 2) and sequencing.

### Cloning of repair arms by Gibson assembly

Each pACRISPR derivative containing a cloned spacer was digested with XbaI and XhoI for 3 hours at 37°C, following a purification step. The upstream and downstream regions of the gene to be deleted (each one with a size between 600 and 1,200 bp) were amplified by PCR using 20–25 bp overlap primers stated in Table 2. Both linearized vector and repair arms were mixed with the NEBuilder HiFi DNA assembly master mix and incubated at 50°C for 1 hour, following the manufacturer’s instructions (New England Biolabs). The purified Gibson assembly product was electroporated into *E. coli,* and confirmation of the plasmids harboring the repair arms was carried out by restriction endonucleases and sequencing of these regions to confirm that no mutations were present.

### Cell transformation

Electrocompetent cells of *E. coli* were prepared as described previously (38). For *Burkholderia* strains requiring induction of gene expression from the *araB* promoter, L-arabinose was added at a final concentration of 2% when cells grown in LB medium reached an OD_640nm_ of 0.8–1.0 and further grown for 3 hours before preparing them as electrocompetent cells. For electrocompetent cells’ preparation, a volume of 100 mL of culture was transferred to a centrifuge tube and cooled in ice for 15 minutes, followed by centrifugation at 5,000 × *g* for 5 minutes at 4°C. The supernatant was removed, and the cell pellet was washed three times with 40 mL ice-cold distilled sterile H_2_O, followed by two washing steps with 20 mL of 10% ice-cold glycerol. In the end, the cell pellet was resuspended in 1 mL of 10% glycerol, and 50 µL aliquots were immediately frozen at −80°C. Electroporation was performed using the BioRad Gene Pulser II system with the following parameters: 25 μF, 2.5 kV, and 200 Ω for *Burkholderia* and 25 μF, 2.5 kV, and 400 Ω for *E. coli*. Cells were recovered in the LB medium for 1 hour (*E. coli*) or 4 hours (*Burkholderia*) before being plated in a selective medium. Triparental conjugation was performed as described previously (40) using plasmids pRK600 or pRK2013 as helper plasmids.

### Genome editing and mutant screening

The general strategy to edit the genome of *B. multivorans* strains was to electroporate at least 1 μg of the pACRISPR-derivative with the spacer and repair arms into electrocompetent cells already harboring pCasPA plasmid. After colony-forming units were developed under kanamycin selection, colony PCR to all colonies, or randomly selected ones, was performed. Here, the primers were selected to amplify the region upstream and downstream of the repair arms present in the chromosome but not from the pACRISPR derivative. After plasmid curing in the desired mutants, a second set of primers (Table 2) was used to confirm gene deletion.

### Plasmid curing after genome editing

*B. multivorans* ATCC 17616-derived deletion mutants carrying pCasPA and/or pACRISPR-Km derivatives were cured by plating cultures’ serial dilutions in LB agar supplemented with 10% sucrose and incubated at 37°C for 24–48 hours. Random colonies were tested for growth in LB agar plates supplemented with 200 μg/mL of tetracycline or 500 μg/mL of kanamycin, depending on the plasmid to be used. For *B. multivorans* P0213-1 mutant derivatives, stationary phase cultures of the previous day were passed into a new LB liquid medium (1:30 dilution) and incubated at 18°C–20°C with agitation for the next 24 hours. After each passage, serial dilutions were plated onto LB agar until colonies were formed. Random colonies were then streaked onto LB plates with or without antibiotics to select the ones that had lost the plasmid.

### Mutant complementation

To complement the *B. multivorans* P0213-1 *ΔbceF* mutant, plasmid pLM127-13 expressing the *bceF* gene was mobilized from *E. coli* to *B. multivorans* by triparental conjugation using the helper plasmid pRK2013. Transconjugants were selected on YEM agar plates containing 200 μg/mL of chloramphenicol and 40 μg/mL of gentamicin.

### Growth assay in the presence of L-arabinose

*B. multivorans* ATCC 17616 cultures were grown in microtiter plates with LB supplemented with 200 μg/mL of tetracycline and with or without L-arabinose at 37°C with shaking for 3 s every 30 minutes, and the OD_640nm_ was measured for 48 hours in a microplate reader. Two independent experiments, each containing six technical replicates, were performed.

### Mucoid phenotype assessment

Exopolysaccharide production was assessed based on the visual inspection of the mucoidy of colonies grown in yeast extract-mannitol agar medium (0.5 g/L yeast extract, 4 g/L D-mannitol, and 2% agar) (41) for 48 hours at 37°C.

### Siderophore production

Siderophore production was determined by the modified Chrome Azurol S (CAS) agar diffusion assay (42). Bacterial cultures were grown in LB liquid medium at 37°C, 250 rpm orbital agitation, for 17 hours. Cultures were diluted to an OD_640nm_ of 1, and 5 µL spots were inoculated onto CAS-MM agar plates (41). Plates were incubated at 37°C and yellow halos were measured after 72 hours.

### Plate-based motility assay

For motility estimation, overnight LB bacterial cultures (5 μL) were inoculated on the agar surfaces of swimming plates and incubated statically right-side up at 37°C for 24 hours followed by colony halo diameter determination. Swimming plates were prepared with an SM medium containing 0.3% (wt/vol) Noble agar (Difco). Two independent experiments, each containing six technical replicates, were performed.

### Microscopy analysis

*B. multivorans* ATCC 17616::*gfp* was grown overnight at 37°C with 200 rpm agitation. To visualize GFP production, images were acquired on a Zeiss Axioplan fluorescence microscope equipped with the AxioCam 503C camera and controlled with software ZEN 3.1 (blue edition) (Zeiss). Images obtained by fluorescence microscopy and light microscopy have a magnification of 1,000×. Images of bacterial colonies were made with a mobile phone’s digital camera.

## Data Availability

Constructed plasmid vectors deposited at Addgene are pACRISPR-KM (#203587), pACRISPR-CM (#203588), and pACRISPR-TP (#203589). Other vectors obtained from Addgene were pCasPA and pACRISPR. The sequences of pBBR1MCS, pUC-TP, pK19mob, and pRK2013 are available at GenBank. Genome sequence assembly of *B. multivorans* P0213-1 (VC7495) was submitted to GenBank with the number GCA_031575995.1.

## SUPPLEMENTAL MATERIAL

Supplemental figures aem.02250-23-s0001.docx Supplemental Figures S1 to S7.

ASM does not own the copyrights to Supplemental Material that may be linked to, or accessed through, an article. The authors have granted ASM a non-exclusive, world-wide license to publish the Supplemental Material files. Please contact the corresponding author directly for reuse.
