# Supplementary material for: Genomic editing in Burkholderia multivorans by CRISPR/Cas9
Source: Appl Environ Microbiol. 2024 Feb 2;90(2):e02250-23. doi: 10.1128/aem.02250-23 (PMC10880607; doi:10.1128/aem.02250-23)
Supplement: Supplemental figures — S1 to S7. [file aem.02250-23-s0001.docx]

**Genomic editing in *Burkholderia multivorans* by CRISPR/Cas9**

**Mirela R. Ferreira^1,2^, Vasco Queiroga^3^, and Leonilde M. Moreira^1,2,3#^**

^1^iBB- Institute for Bioengineering and Biosciences, Instituto Superior Técnico, Universidade de Lisboa, Av. Rovisco Pais, 1049-001 Lisboa, Portugal

^2^Associate Laboratory i4HB-Institute for Health and Bioeconomy, Instituto Superior Técnico, Universidade de Lisboa, Av. Rovisco Pais, 1049-001 Lisboa, Portugal

^3^Department of Bioengineering, Instituto Superior Técnico, Universidade de Lisboa, Av. Rovisco Pais, 1049-001 Lisboa, Portugal

^#^Corresponding author

E-mail address: [lmoreira@tecnico.ulisboa.pt](mailto:lmoreira@tecnico.ulisboa.pt)

**Supplemental Figures**


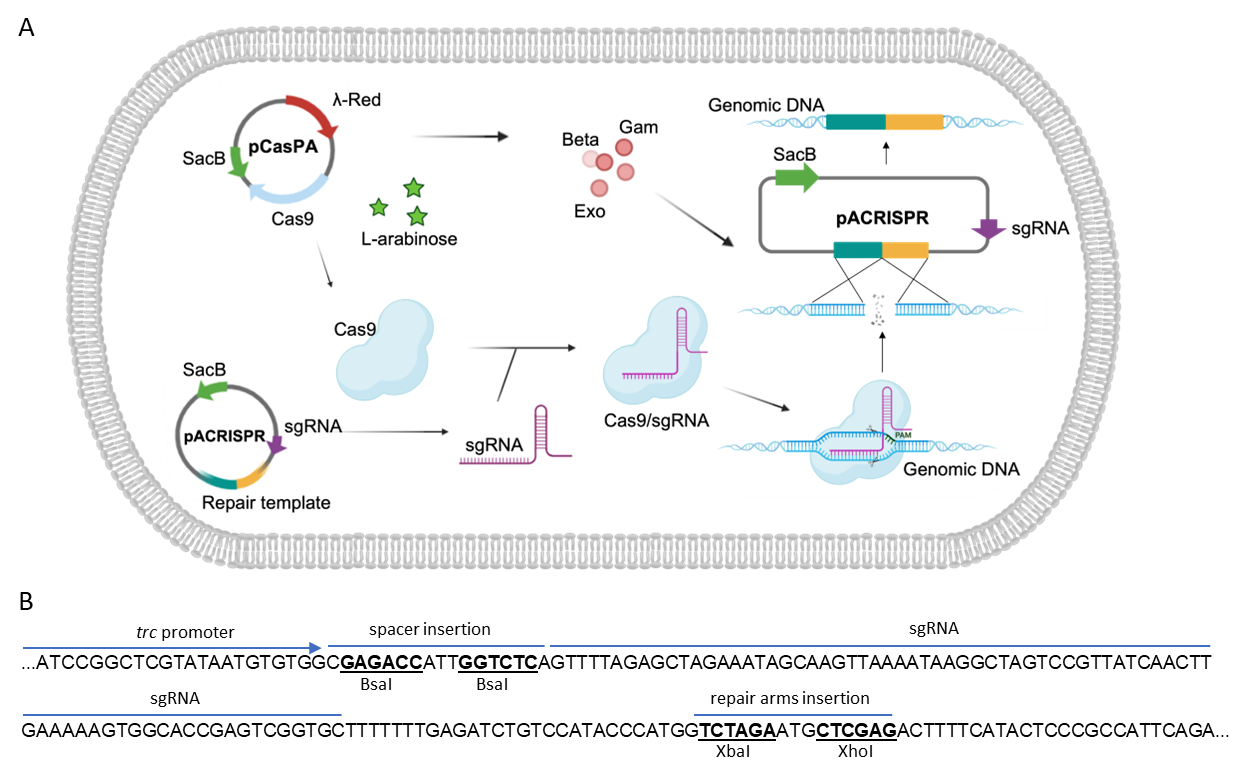


**Figure S1-** **Overview of the CRISPR/Cas9 genome editing tool developed by Chen et al. (2018)**. (A) Plasmid pCasPA contributes to the expression of genes encoding the Cas9 and λ-Red system proteins after induction with L-arabinose. Plasmid pACRISPR leads to the transcription of the single guide RNA (sgRNA) and carries the repair arms. Loading the Cas9 with the sgRNA results in a double-strand break within the desired genome locus, which stimulates the repair mechanism of homologous recombination between the genome and the repair arms present in pACRISPR plasmid assisted by the λ-Red system proteins. Both plasmids contain the counter-selectable *sacB* gene, which works as a lethal gene in the presence of sucrose, allowing for the identification of cells where plasmids were lost. Selection markers in pCasPA and pACRISPR confer resistance to tetracycline and carbenicillin, respectively. (B) Sequence of the pACRISPR plasmid showing the restriction endonucleases sites for cloning of the spacer and repair arms template. (Created with BioRender.com).


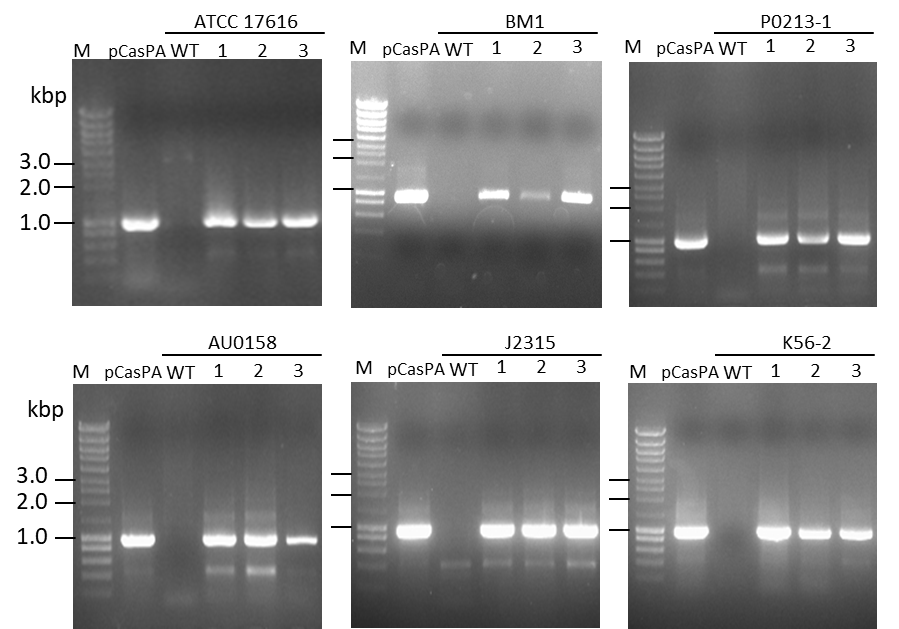


**Figure S2-** **Transformation of pCasPA into *Burkholderia* strains**. Gel electrophoresis shows the amplification of a 936-bp (for BM1) or 937-bp (for the remaining strains) internal fragment of the *cas9* gene in the transformed colonies, but not in the wild-type strains. Vector pCasPA was used as a positive control. The tested strains are *B. multivorans* ATCC 17616, BM1, and P0213-1; *B. dolosa* AU0158; and *B. cenocepacia* J2315 and K56-2.


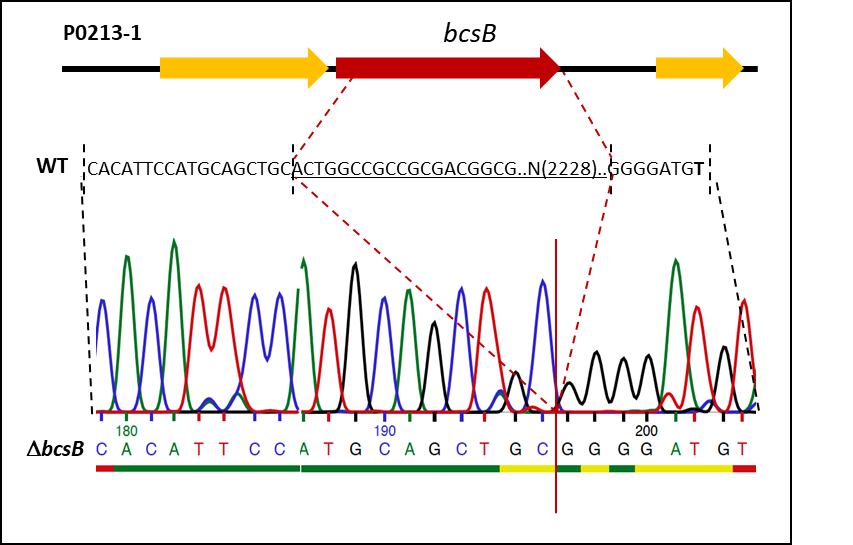


**Figure S3-** **Confirmation of *bcsB* gene deletion in *B. multivorans* P0213-1.** The deleted region comprises 2247 bps and is underlined.

**
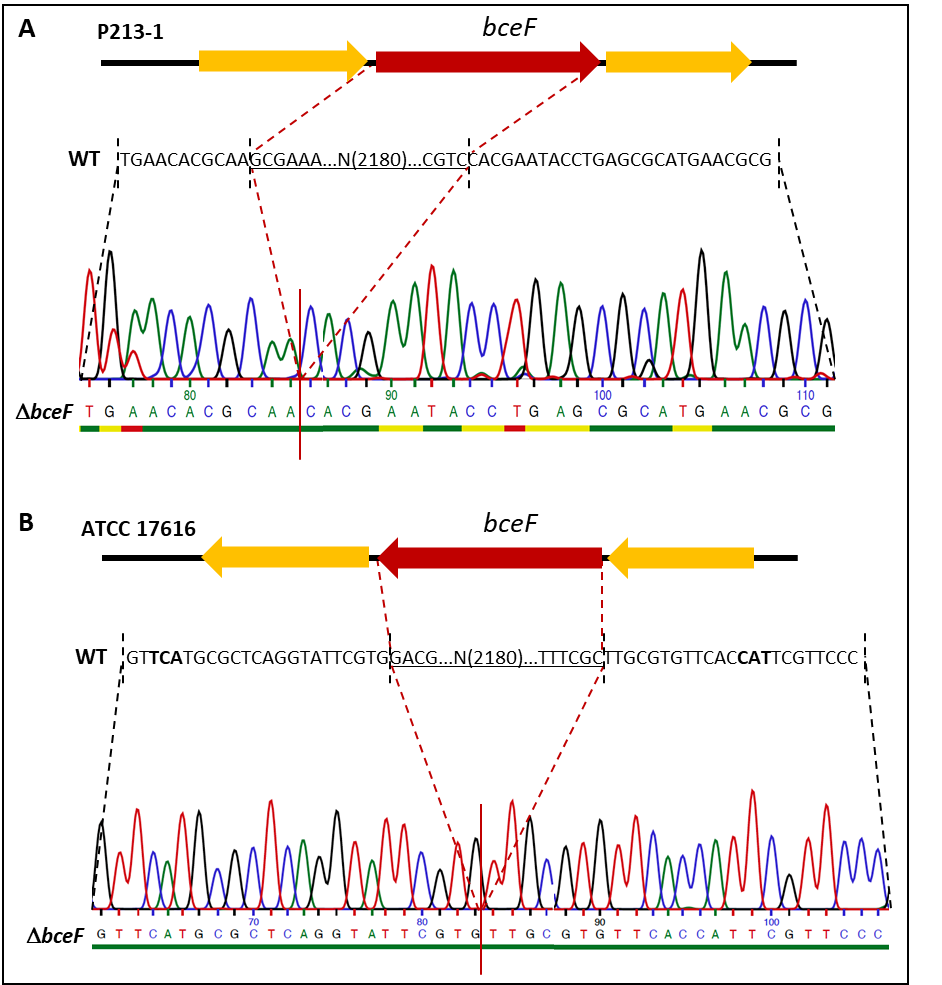
**

**Figure S4-** **Confirmation of *bceF* gene deletion in *B. multivorans* P0213-1 (A) and ATCC 17616 (B).** The deleted region comprises 2190 bps and is underlined. For ATCC 17616, the sequence of the reverse strand is shown.

**
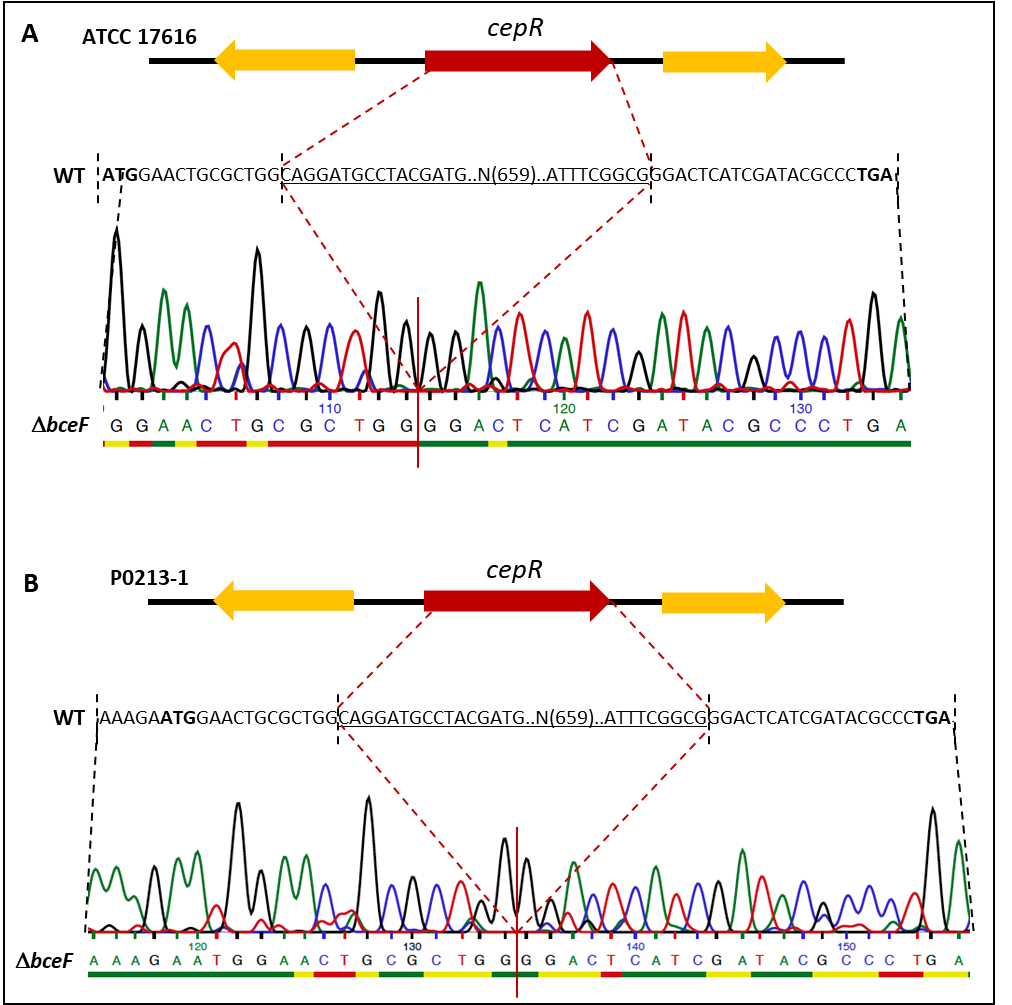
**

**Figure S5-** **Confirmation of *cepR* gene deletion in *B. multivorans* ATCC 17616 (A) and P0213-1 (B).** The deleted region comprises 684 bps and is underlined. The initiation and stop codons of *cepR* coding sequence are in bold.


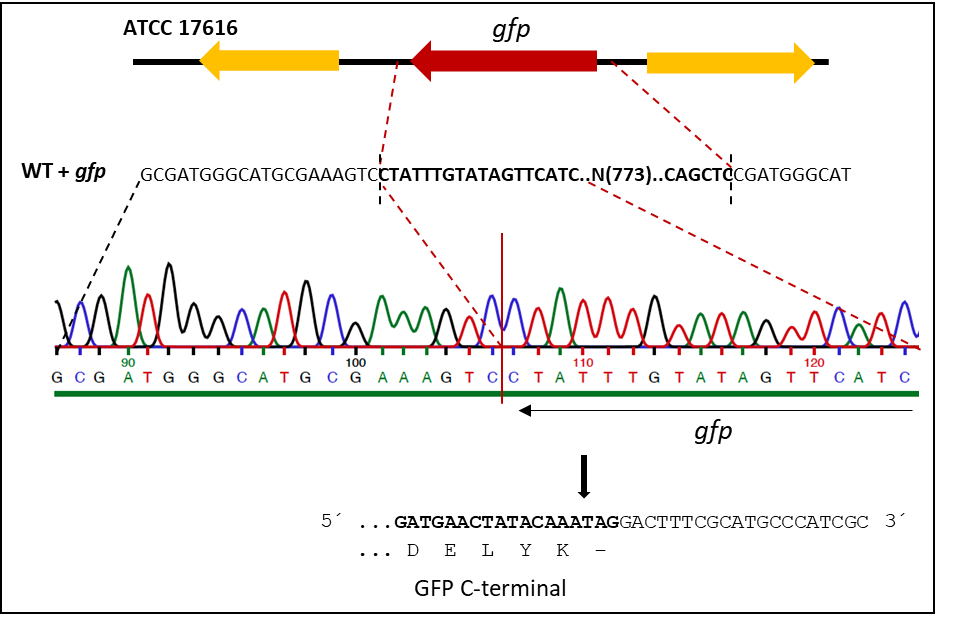


**Figure S6-** **Confirmation of *gfp* gene insertion in *B. multivorans* ATCC 17616.** The inserted region of 797 bps comprises the promoter and *gfp* gene coding sequence and is represented in bold. The last five amino acids of GFP are showed.


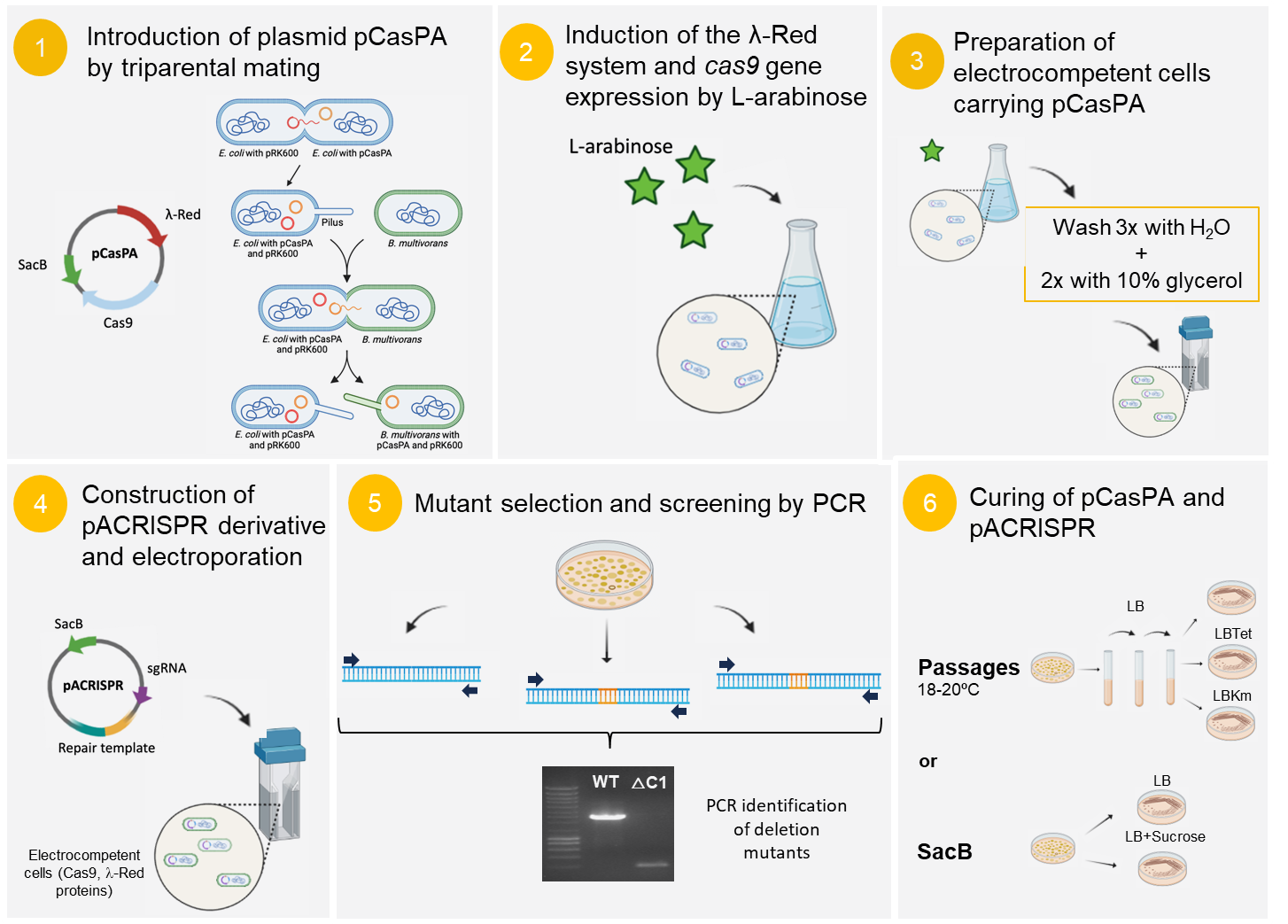


**Figure S7-** **Overview of the experimental steps for CRISPR/Cas9-mediated genome editing in *Burkholderia multivorans***. The bacteria are first transformed with pCasPA (step 1), then Cas9 and λ-Red system are induced and electrocompetent cells are prepared (steps 2 and 3). These cells are transformed with a pACRISPR-derivative which encodes the sgRNA and has the repair arms (step 4). Obtained colonies are then screened for deletion/insertion of the gene of interest by PCR (step 5), followed by plasmid curing by the activity of *sacB* gene in the presence of sucrose or by repeated passages of cells grown at low temperature (step 6). The choice of the plasmid curing method is strain dependent. (Created with BioRender.com).
